# Validation of an enzyme-linked immunosorbent assay (ELISA) for quantification of endostatin levels in mice as a biomarker of developing glomerulonephritis

**DOI:** 10.1371/journal.pone.0220935

**Published:** 2019-08-12

**Authors:** Jacqueline Wallwitz, Petra Aigner, Elisabeth Gadermaier, Eva Bauer, Emilio Casanova, Anton Bauer, Dagmar Stoiber

**Affiliations:** 1 The Antibody Lab GmbH, Vienna, Austria; 2 Ludwig Boltzmann Institute for Cancer Research, Vienna, Austria; 3 Institute of Physiology, Center for Physiology and Pharmacology, Medical University of Vienna, Vienna, Austria; 4 Institute of Pharmacology, Center for Physiology and Pharmacology, Medical University of Vienna, Vienna, Austria; 5 Department of Pharmacology, Physiology and Microbiology, Division Pharmacology, Karl Landsteiner University of Health Sciences, Krems, Austria; The Pennsylvania State University, UNITED STATES

## Abstract

Endostatin, the C-terminal fragment of type XVIII collagen, was shown to be one of the most potent endothelial cell-specific inhibitors of angiogenesis. As altered circulating endostatin concentration is associated with impaired kidney function, new tools for measuring endostatin in rodents may be helpful to further investigate and understand its role within kidney disease progression. A novel and commercially available ELISA for the quantification of mouse and rat endostatin was developed and validated according to international quality guidelines including the parameters specificity, robustness, accuracy, dilution linearity, precision, limit of detection (LOD) and lower limit of quantification (LLOQ). Endostatin and blood urea nitrogen (BUN) concentration were measured in mice with a glomerulonephritis phenotype. The validation revealed that within the range of 0.5–32 nmol/L the immunoassay is robust and highly specific for the measurement of rodent endostatin with high sensitivity (LOD 0.24 nmol/L, LLOQ 0.5 nmol/L) and good reproducibility (intra- and inter-assay CV <10%). Also accuracy and dilution linearity were within the range of acceptance. *BCL2* transgenic and *ETV6/RUNX1;BCL2* double transgenic mice develop a glomerulonephritis phenotype over time, which was displayed by staining of kidney sections. Even before full manifestation of disease serum endostatin concentration rises significantly, whereas BUN levels just slightly increase. This newly developed and commercially available ELISA provides a reliable and accurate tool for the quantification of mouse and rat endostatin and may give new perspectives in the investigation of the role of endostatin as an important and early biomarker for reduced kidney function. Measurement of endostatin concentration is recommended to be used as a superior biomarker for chronic kidney disease compared to BUN.

## Introduction

Decreased kidney function may have a wide range of reasons like infections, toxins, genetic disorders, metabolic dysfunction like type 2 diabetes or autoimmune diseases. There are various underlying pathological changes, which may result in acute kidney injury (AKI) or promote the development of chronic kidney disease (CKD). AKI is defined as a sudden reduction in the glomerular filtration rate (GFR) and renal output, which results in the accumulation of nitrogenous waste [1], whereas CKD is characterized by structural or functional abnormalities of the kidney with implications for health over a time period for at least three months [2]. Albuminuria, blood urea nitrogen (BUN) and estimated glomerular filtration rate (eGFR) are the classical guideline-endorsed biomarkers for the classification of CKD and are strong predictors of renal disease progression and morbidity in human, but also in rodents. Currently, there is huge effort undertaken to define new biomarkers or biomarker panels for prognosis and diagnosis of kidney disease, as well as for a deeper understanding of renal pathology and for the identification of potential therapeutic targets [3].

Endostatin could be one of these promising markers, as it has gained increasing interest over the last years. Of note, endostatin was already discovered 1997 by O’Reilly and colleagues and is a 20 kDa inhibitor of endothelial cell proliferation *in vitro* and a potent inhibitor of angiogenesis and tumor growth *in vivo* [4]. It is the C-terminal fragment of type XVIII collagen and emerges mainly during extracellular matrix remodeling [5]. Recent studies have shown that altered expression [6–8] and increased circulating endostatin concentration [9,10] are associated with impaired kidney function. Importantly, endostatin could be also used as a prediction marker for AKI in critically ill patients [11].

*Vav-BCL2* transgenic mice (BCL2^tg^) are prone to suffer from follicular lymphoma with age and to develop kidney disease, i.e. glomerulonephritis of an autoimmune type [12]. Previously a synergism between the ETV6/RUNX1 fusion product, resulting from the t(12;21) translocation in humans, and the anti-apoptotic molecule BCL2 was identified using a new mouse model [13]. After intercrossing *ETV6/RUNX1* and *BCL2* single transgenic mice the resulting double transgenic animals (E/R^tg^;BCL2^tg^) harbored elevated B cell numbers and autoreactive immunoglobulin titers when compared to BCL2^tg^ mice. This resulted in profound deposition of immune complexes in glomeruli and accelerated development of immune complex glomerulonephritis [13].

In order to further identify the biological function of endostatin in different mouse models during kidney pathogenesis there is a need to reliably and reproducibly measure endostatin concentration in serum and plasma. As there is no high-quality quantification tool available, this study presents the development, validation and application of an enzyme-linked immunosorbent assay (ELISA) for measuring rodent endostatin levels, which is also available commercially. Biological relevance for endostatin as an early biomarker could be shown with the determination of endostatin concentration in *BCL2* transgenic and *ETV6/RUNX1;BCL2* double transgenic mice with impaired kidney function.

## Materials and methods

### Mice

All animal experiments were carried out in strict accordance with recommendations of the Good Scientific Practice Guidelines of the Medical University of Vienna. The protocol was approved by the Ethics Committee for Laboratory Animal Research of the Medical University of Vienna and by the Austrian Federal Ministry of Science, Research and Economy (Permit Number BMWF-66.009/0279-II/3b/2012). All efforts were made to minimize animal suffering in all procedures.

CD19+ B cell specific *ETV6/RUNX1*-expressing (E/R^tg^) [14], *Vav-BCL2* transgenic (BCL2^tg^) [15] and E/R^tg^;BCL2^tg^ [13] mice were maintained on C57BL/6 background, wild type C57BL/6 littermates were used as controls. *NOD*.*Cg-Prkdc*^*scid*^ *Il2rg*^*tm1WjI*^*/SzJ* (NSG) mice were used at the age of 8–10 weeks for transplantation experiments, where 10^6^ bone marrow cells were injected via tail vein. All mice were bred at the Medical University of Vienna and housed under SPF conditions with temperatures ranging from 21–23°C in isolated ventilated cages (≤ 5 mice per cage; SmartFlow and EasyFlow, Tecniplast, Buguggiate, Italy) with a 12 hr light cycle (7 am– 7 pm). Cages contained standard bedding material and mice were fed autoclaved standard laboratory chow (commercial control diet for mice; ssniff R/M-H, Soest, Germany, Code V1536-000). Animals were provided with enrichment to ensure their welfare and at baseline were free of pathogens and healthy. The investigators were not blinded to the groups of mice used in this study and no exclusion criteria were used during analysis. Unless otherwise indicated in all experiments, male as well as female mice were used.

Mice were sacrificed and analyzed at defined time points or at first signs of disease according to ethical guidelines. Veterinarian and animal technicians especially trained in courses accredited by the Federation of European Laboratory Animal Science Associations (FELASA) monitored the welfare and health status of mice daily. Humane endpoints included abdominal distension, ruffled fur, labored breathing, immobility or reduced food intake. Once animals reached humane endpoint criteria they were immediately sacrificed humanely using cervical dislocation. Owing to the lymphoma and glomerulonephritis phenotype of the two mouse groups–BCL2^tg^ and E/R^tg^;BCL2^tg^—<5% of animals were humanely euthanized, no animals died before meeting the criteria for euthanasia. Blood was collected and subsequently centrifuged. Sera were stored at -20°C until further use. Serum levels of urea were measured using Reflotron-based test strips (Roche Applied Science, Penzberg, Germany). Blood urea nitrogen was calculated using the following formula: urea (mg/dL) x 0.467 = BUN (mg/dL). Formalin-fixed, paraffin-embedded kidney sections were stained with Periodic Acid Schiff (PAS) according to standard procedures.

All animal experiments were carried out in strict accordance with the recommendations and requirements as defined by the Federation of European Laboratory Animal Science Associations (FELASA). The protocol was reviewed by the Ethics Committee for Laboratory Animal Research of the Medical University of Vienna and approved by the Austrian Federal Ministry of Science, Research and Economy (Permit Number BMWF-66.009/0279-II/3b/2012), where the 3Rs have previously been addressed. All efforts were made to minimize animal suffering in all procedures.

### ELISA protocol

The mouse and rat endostatin ELISA (BI-20742MR, Biomedica, Vienna, Austria) is based on a sandwich type format, where the affinity purified polyclonal goat antibody is immobilized on a 96-well microtiter plate. The detection antibody is an affinity purified polyclonal goat antibody conjugated to horseradish peroxidase and diluted in a peroxidase-stabilizing buffer system.

The assay includes seven standards (0 (STD1), 1 (STD2), 2 (STD3), 4 (STD4), 8 (STD5), 16 (STD6) and 32 (STD7) nmol/L) and one control, which contain recombinant mouse endostatin diluted in a protein-based buffer system. Standards, control and samples are pre-diluted 1+50 in a tube using the protein-based assay buffer. 50 μl of these-predilutions are pipetted into the pre-coated plate and subsequently 50 μl of detection antibody solution is added per well. The plate is covered with an adhesive strip and incubated for two hours at room temperature. Each well is aspirated and washed five times before adding 100 μl of 3,3’,5,5’-tetramethylbenzidine (TMB) substrate solution to each well. After 30 minutes of incubation at room temperature 50 μl of the stop solution is added and the optical density (OD) is obtained with a spectrophotometer using 450 nm as reference and 630 nm as correction wavelength (BioTec, Winooski, VT, USA). The standard curve is fitted using the 4PL algorithm. Concentration of samples and controls are calculated based on the standard curve. Our assay has been commercialized by Biomedica (BI-20742MR, Biomedica, Vienna, Austria).

### Precision

For intra-assay precision within one run a 5-fold determination of standards and samples was performed. For reproducibility (inter-assay precision) the same set of samples was used and measured in at least three different runs, with different equipment, different operators, and on different days. The coefficient of variation (CV) in % was calculated.

### Specificity, cross-reactivity, accuracy and dilution linearity

For specificity testing an at least 5-fold surplus of coating antibody (competitor) was added to tested samples. Percentage of competition was calculated. To determine cross-reactivity and interferences in the ELISA various other recombinant proteins were diluted to a concentration of 50 ng/ml in 10 mM PBS supplemented with 1% BSA and 0.05% Tween and measured according to the assay protocol. Following recombinant proteins were used: recombinant mouse proteins comprising angiopoietin-2 (R&D Systems, Minneapolis, USA), FGF-23 (R&D Systems, Minneapolis, USA), gremlin (R&D Systems, Minneapolis, USA), periostin (R&D Systems, Minneapolis, USA), TIM-1 (R&D Systems, Minneapolis, USA), TNFalpha (R&D Systems, Minneapolis, USA) and vanin (custom synthesized protein), as well as recombinant rat proteins including CTGF (R&D Systems, Minneapolis, USA) and NT-proANP (custom synthesized peptide) and recombinant human proteins FGF-3 (R&D Systems, Minneapolis, USA), FGF-19 (R&D Systems, Minneapolis, USA), FGF-21 (R&D Systems, Minneapolis, USA), FGF-23 (R&D Systems, Minneapolis, USA), Gremlin (R&D Systems, Minneapolis, USA) and Coll 2–1 (custom synthesized peptide). For accuracy measurements, samples were spiked with different concentration of recombinant endostatin. Spike recovery was calculated as the ratio of measured total endostatin concentration over the expected value. Spiked and unspiked samples were diluted 1:2, 1:4 and 1:8 with assay buffer and dilution linearity was calculated as the ratio of measured diluted endostatin concentration multiplied with the dilution factor over the undiluted endostatin concentration.

### LOD and LLOQ

For calculation of the limit of detection (LOD) the lowest standard was determined 10-fold and three times the standard deviation was added. For the lower limit of quantification (LLOQ) Standard 2 (1 nmol/L) was further diluted with assay buffer 1:2, 1:4 and 1:8. For the determination of LLOQ three experiments with a five-fold determination per dilution were performed.

### Stability of recombinant and endogenous endostatin

Serum samples as well as recombinant mouse standards in protein based buffer were used for analyte integrity during various freeze-thaw cycles. Each cycle comprises one hour freezing at -25°C following one hour thawing at room temperature. After last thawing, samples and standards were stored at -25°C and measured together within one run.

### Statistical analysis

Data analysis was performed using Excel (Microsoft Corp., Redmond, USA) and GraphPad Prism (GraphPad Software Inc., La Jolla, USA). Significance between two groups was calculated using unpaired t-test.

## Results

### Specificity, cross-reactivity, sensitivity and precision of the endostatin ELISA

Specificity is the ability to assess unequivocally the analyte and is one of the most important characteristics for reliable and reproducible measurements. Both used antibodies need to predominantly bind to mouse and rat endostatin, and should interact neither with other unspecific components of the sample matrix nor with structurally related compounds. Signal reduction after adding the competitor was 100% (99–100%) for mouse serum (n = 8), 98% (95–100%) for mouse plasma (n = 8), and 100% (99–100%) for rat serum samples (n = 5) (Table 1, S1 Table), respectively, which indicates that the generated signals are highly specific for endostatin in all tested matrices.

**Table 1:** Specificity of endostatin ELISA for mouse and rat samples.

Samples (n)	Mean conc. [nmol/L] w/o competitor	Mean conc. [nmol/L] with competitor	Mean competition %
**Mouse serum (8)**	4.0	0.0	100
**Mouse plasma (8)**	2.9	0.1	98
**Rat serum (5)**	1.5	0.0	100

Conc., concentration; w/o, without.

A further approach to prove specificity of a sandwich immunoassay is cross-reactivity testing. The measured values of six human (Coll 2–1, FGF-3, FGF-19, FGF-21, FGF-23 and Gremlin), seven mouse (ANG-2, FGF-23, Gremlin, Periostin, TIM-1, TNF alpha, Vanin-1) and two rat (CTGF, NT-proANP) proteins were below background (S1 Table), although the used concentration of 50 ng/ml was more than 76 fold above the highest mouse endostatin standard with 0.65 ng/ml. Therefore, neither cross-reactivity, nor interferences with these proteins (summarized in Table 2) in the endostatin ELISA were observed.

**Table 2:** No cross-reactivity with or interference of other proteins.

Recombinant human	Recombinant mouse	Recombinant rat
Coll 2–1	ANG-2	CTGF
FGF-3	FGF-23	NT-proANP
FGF-19	Gremlin	
FGF-21	Periostin	
FGF-23	TIM-1	
Gremlin	TNF alpha	
	Vanin-1	

The precision of an analytical procedure expresses the closeness of agreement (degree of scatter) between a series of measurements obtained from multiple testing of the same homogeneous sample under the same conditions and is expressed as the coefficient of variation (CV in percent) (EMA guideline Rev. 1 Corr. 2, 2011). Repeatability (intra-assay precision) describes the precision under the same operating conditions (Table 3, S1 Table).

**Table 3:** Intra- and inter-assay precision of mouse/rat endostatin ELISA.

	Samples	Mean measured conc. [nmol/L]	CV [%]
**Intra-assay precision (n = 5)**	**STD2**	0.98	9
	**STD7**	31.98	2
	**Serum 1**	3.2	3
	**Serum 2**	3.3	3
	**Plasma 1**	5.1	2
**Inter-assay precision (n = 5)**	**STD2**	1.01	10
	**STD7**	31.99	2
	**Serum 1**	3.1	5
	**Serum 2**	3.1	6
	**Plasma 1**	5.1	5

STD2 and STD7, standard 2 and standard 7 (recombinant endostatin); conc., concentration

The low standard (STD2) has an average endostatin concentration of 0.98 ± 0.1 nmol/L (CV = 9%, n = 5) and the highest standard (STD7) of 31.98 ± 0.5 nmol/L (CV = 2%, n = 5), respectively. The CV % for both low serum samples was 3% (3.2 ± 0.1 and 3.3 ± 0.1 nmol/L, n = 5 each) and for the low mouse plasma sample it was 5.1 ± 0.1 nmol/L (CV = 2%, n = 5) (Table 3). The same set of samples was used for determination of inter-assay precision (reproducibility). Therefore, all possible factors except laboratory were varied, and measurements were carried out over several days. Within the measurements of recombinant endostatin STD2 gave a mean concentration of 1.01 ± 0.1 nmol/L (CV 10%, n = 15) and STD7 of 31.99 ± 0.5 nmol/L (CV 2%, n = 15). For both endogenous serum samples, the CV was 5% and 6%, respectively, and plasma sample had a CV of 5% (Table 3, S1 Table).

The limit of detection (LOD), defined as the calculated concentration of the lowest standard point (STD1) analyzed in 10-fold determinations plus three times the standard deviation, is 0.34 nmol/l for this endostatin ELISA (Table 4, S1 Table).

**Table 4:** Limit of detection.

	Samples	Mean theoretical conc. [nmol/L]	Mean measured conc. [nmol/L]	SD	LOD
**LOD**	**STD1**	0.0	0.13	0.07	0.34

The lower limit of quantification (LLOQ) is defined as the lowest concentration that can be measured with acceptable accuracy (calculated backfit deviates less than 25% from the theoretical concentration) and precision (CV% < 25%). Therefore, STD2 was serially diluted (1:2, 1:4 and 1:8) and measured three times in a 5-fold determination with a CV of 5%, 10% and 27% (Table 5, S1 Table).

**Table 5:** Lower limit of quantification.

	Samples	Mean theoretical conc. [nmol/L]	Mean measured conc. [nmol/L]	CV [%]	Backfit [%]
	**STD2 (1:2)**	0.5	0.44	5	88
**LLOQ**	**STD2 (1:4)**	0.25	0.37	10	146
	**STD2 (1:8)**	0.125	0.16	27	125

SD, standard deviation; LOD, limit of detection; LLOQ, lower limit of quantification

The calculated backfit of measured compared with theoretical concentration was 88%, 146% and 125%. Taking both measurements into account the LLOQ of this assay is defined as 0.5 nmol/l (1:2).

### Robustness of the ELISA

**Fig 1:**
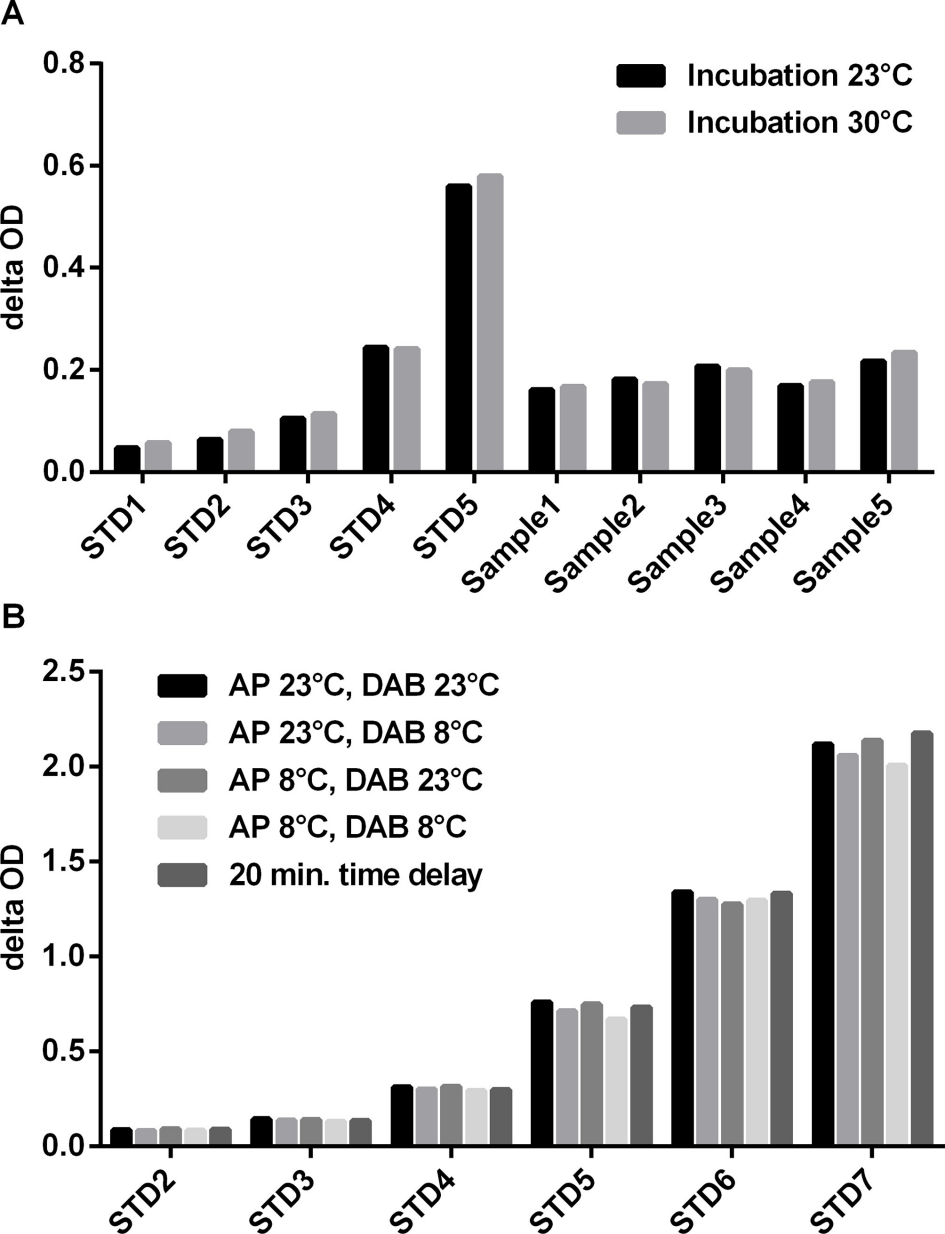
Robustness of the endostatin ELISA. (A) Influence of assay incubation temperature (23°C vs. 30°C) on recombinant endostatin (STD 2–5) and sample OD. (B) Influence of assay buffer and detection antibody solution temperature (23°C and 8°C) as well as 20 minutes delayed pipetting of samples (= drift over plate) on delta OD of recombinant endostatin (STD 1–7).

Robustness of an analytical procedure is defined as the ability to remain unaffected by small variations in method parameters [16]. These parameters could be incubation temperature, temperature of used components or varied incubation time due to time delay during pipetting (Fig 1, S1 Table).

Fig 1A shows the effect of changes in room temperature during incubation. Samples and standards were incubated either at 23°C (as recommended in the package insert) or at 30°C (mimicking a hot summer day). Increased incubation temperature from 23°C to 30°C has no impact (CV < 10%) on OD of samples and standards.

According to the manufacturer´s protocol all ELISA kit components need to be warmed up to room temperature before use. Fig 1B illustrates the influence of different reagent temperature combinations on OD of the standards. There is no significant influence on OD for the usage neither of cold assay buffer nor of cold detection antibody solution or combinations thereof.

A third parameter of robustness, especially for assays with short incubation times, is the drift over plate induced by the time used for pipetting many samples. A robust ELISA is characterized by generating same OD for samples pipetted first and last. Fig 1B shows that there is no change in OD (CV <10%) for samples pipetted with a delay of 20 minutes.

In summary, these experiments for robustness reveal that the assay is unsusceptible to moderate variations in incubation temperature and incubation time.

### Dilution linearity of endogenous and recombinant endostatin

**Fig 2:**
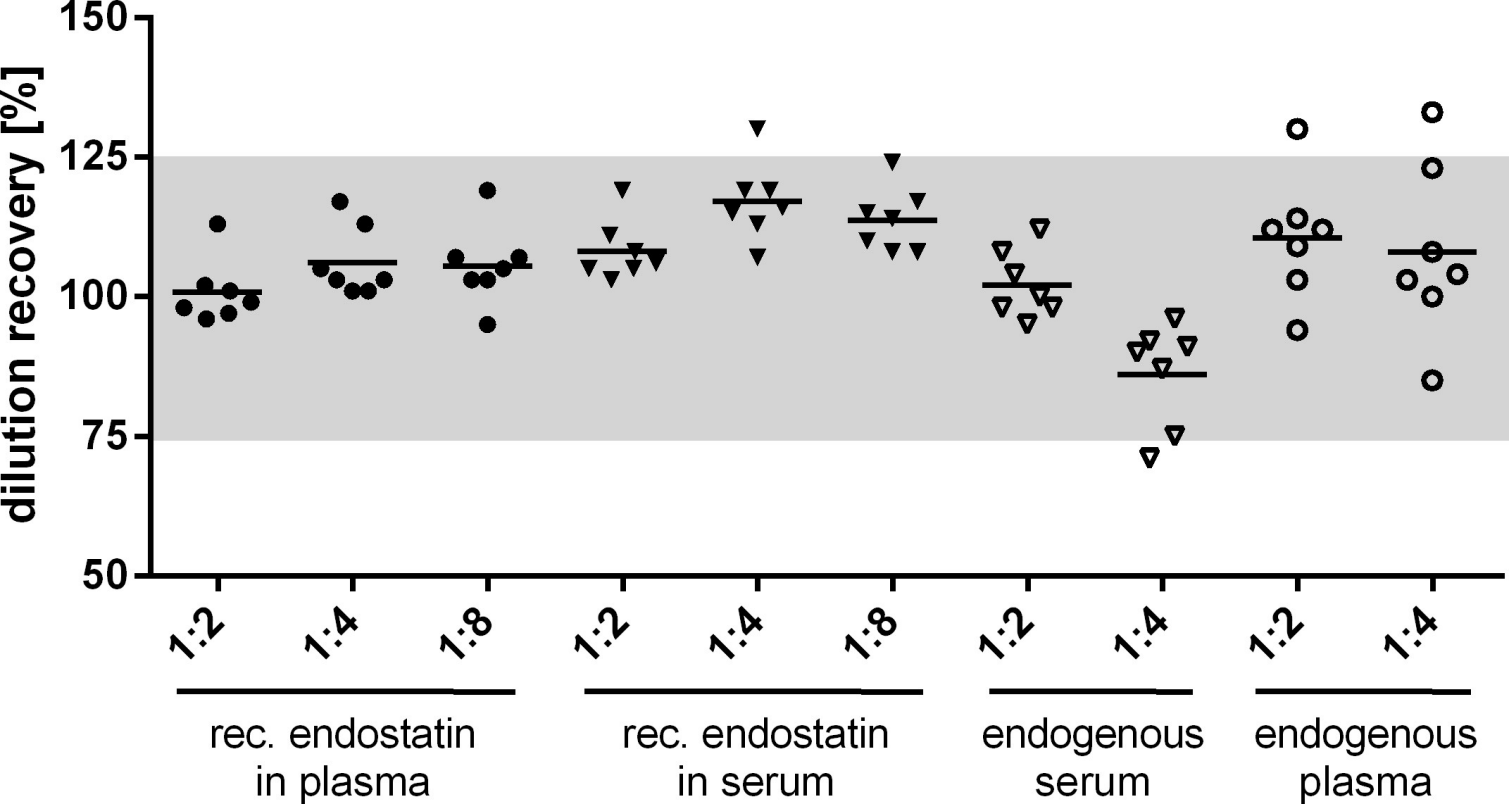
Dilution linearity of recombinant and endogenous endostatin. Dilution recovery in % (y-axis) of recombinant (rec.) mouse endostatin spiked in seven mouse serum and plasma samples and afterwards diluted 1:2, 1:4 and 1:8 with assay buffer and of endogenous endostatin of seven mouse serum and plasma samples diluted 1:2 and 1:4 with assay buffer. Dilution recovery (%) of each sample was calculated and should be within ± 25% (grey area).

Dilution linearity is assessed to ascertain that the employed antibodies show the same binding characteristics to the endogenous analyte and to the calibrator (recombinant mouse endostatin). In order to measure valid sample concentration, the calculated recovery of diluted samples should be in a linear range of 100% ± 25%. For the determination of dilution linearity serum and plasma samples were spiked with 25 nmol/L recombinant endostatin and diluted 1:2, 1:4 and 1:8 (Fig 2, S1 Table).

A mean dilution recovery of recombinant endostatin in serum of 108% (103–119%) for the 1:2 dilution, 117% (107–130%) for the 1:4 dilution and 114% (108–124%) for the 1:8 dilution was calculated. Spiked plasma samples have a mean dilution recovery of 101% (96–113%) for 1:2, 106% (101–113%) for 1:4 and 106% (95–116%) for 1:8 dilution. Endogenous endostatin was diluted 1:2 and 1:4 in serum and plasma samples and calculated mean recovery was 102% (95–112%) and 86% (71–96%) for serum and 110% (94–130%) and 108% (85–133%) for plasma, respectively.

### Accuracy (spike recovery) of the endostatin ELISA

The accuracy of an analytical method describes the closeness of the determined values obtained by the method to the true concentration of the (recombinant) analyte (expressed in percentage). To assess the accuracy, serum as well as plasma samples were spiked with 8 nmol/L and with 25 nmol/L recombinant endostatin (Table 6, S1 Table).

**Table 6:** Accuracy (spike recovery) of mouse/rat endostatin ELISA.

Matrix	n	8 nmol/L rec. endostatin recovery [%]	25 nmol/L rec. endostatin recovery [%]
Serum	7	97 (95–112)	95 (85–111)
Plasma	6	82 (71–89)	86 (62–96)

Rec. endostatin, recombinant endostatin. Mean (range) spike recovery.

For serum, mean recovery was 97% (95–112%) for the spike in the middle, and 95% (85–111%) in the higher calibration range. It was slightly lower for plasma with 82% (71–89%) and 86% (62–96%), respectively.

### Stability of the endogenous and recombinant endostatin

**Fig 3:**
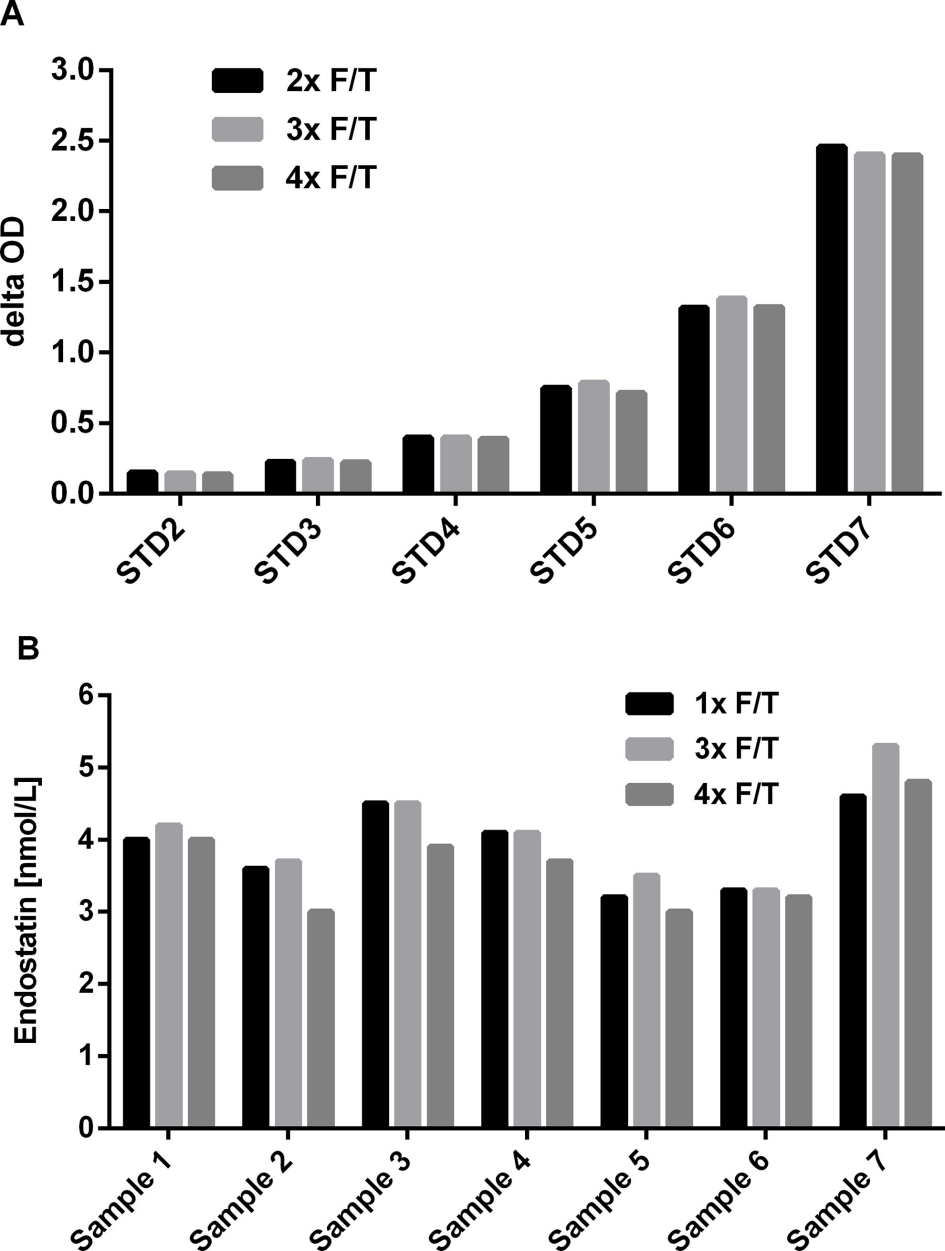
Freeze/thaw stability of recombinant and endogenous endostatin. (A) Delta OD of recombinant endostatin (STD 2–7) and (B) concentration (nmol/L) of endogenous endostatin (sample 1–7) subjected to up to four freeze/thaw (F/T) cycles.

The freeze/thaw stability of undiluted endogenous endostatin and recombinant standards was assessed (Fig 3, S1 Table).

Recombinant mouse endostatin (Fig 3A) and also endogenous endostatin in serum (Fig 3B) was found to be stable for at least four freeze-thaw cycles with a CV between the measurements of 1–4% and 1–10%, respectively.

### Endostatin levels in mice with glomerulonephritis

**Fig 4:**
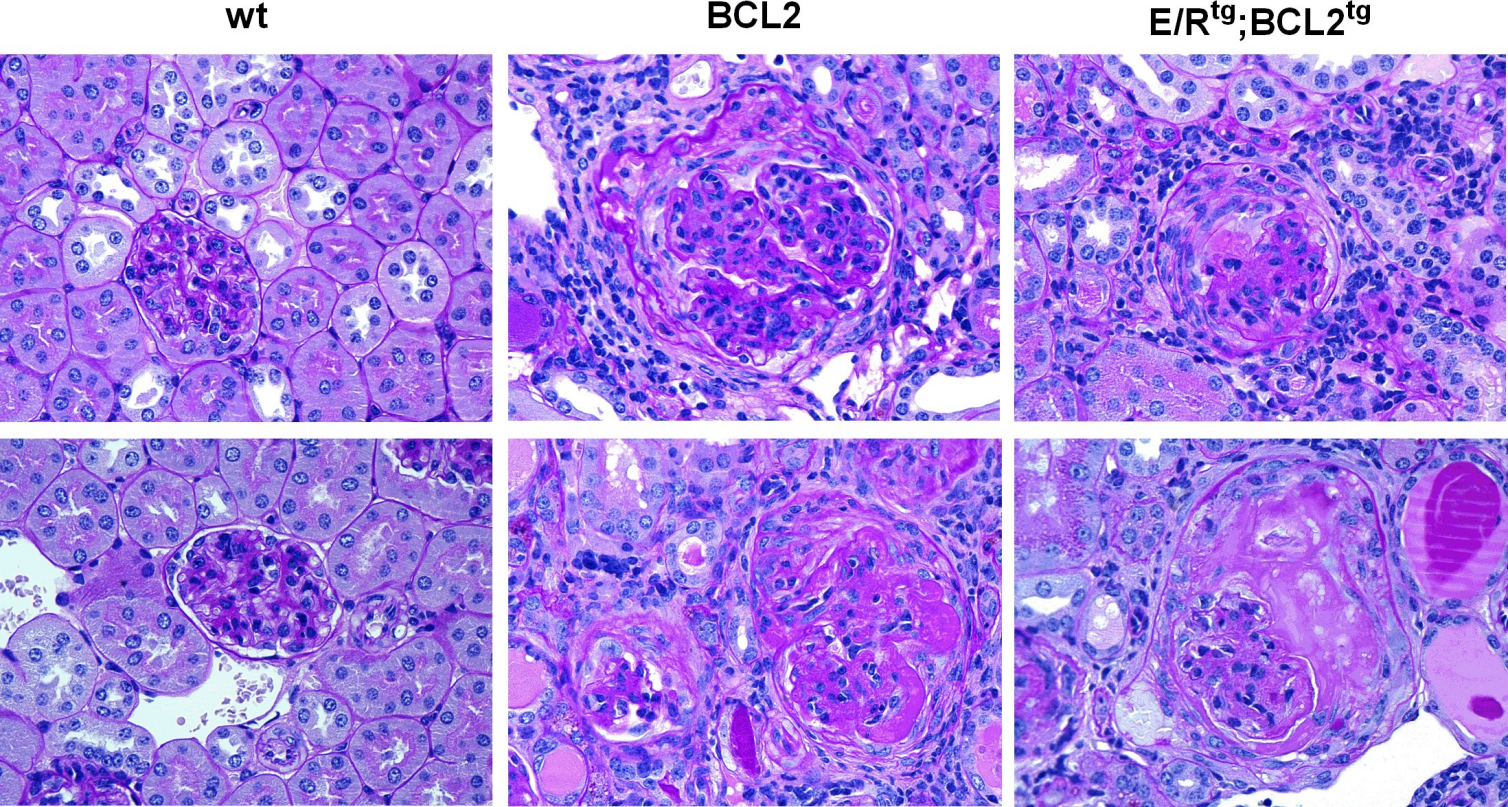
BCL2^tg^ and ETV6/RUNX1^tg^;BCL2^tg^ mice develop glomerulonephritis. Representative pictures of kidney sections of wild type (wt), BCL2^tg^ and E/R^tg^;BCL2^tg^ mice (n = 2 for each genotype) are shown. Pictures in lower panels (middle and right) correspond to further progressed glomerulonephritis than in upper panels (middle and right). Periodic Acid Schiff (PAS) staining of the kidney sections indicates enlarged glomeruli and crescents in BCL2^tg^ and E/R^tg^;BCL2^tg^ mice.

We have generated a mouse model exhibiting immune complex glomerulonephritis by intercrossing *ETV6/RUNX1* transgenic (E/R^tg^) and *Vav-BCL2* transgenic (BCL2^tg^) mice [12]. In Fig 4, Periodic Acid Schiff (PAS) staining of kidney sections illustrates the diseased kidney phenotype of BCL2^tg^ and E/R^tg^;BCL2^tg^ mice with milder occurrence in the upper pictures and further developed disease with enlarged glomeruli and crescents in the lower panel. E/R^tg^ mice have a similar phenotype as wild type mice, and develop glomerular pathology only with age.

**Fig 5:**
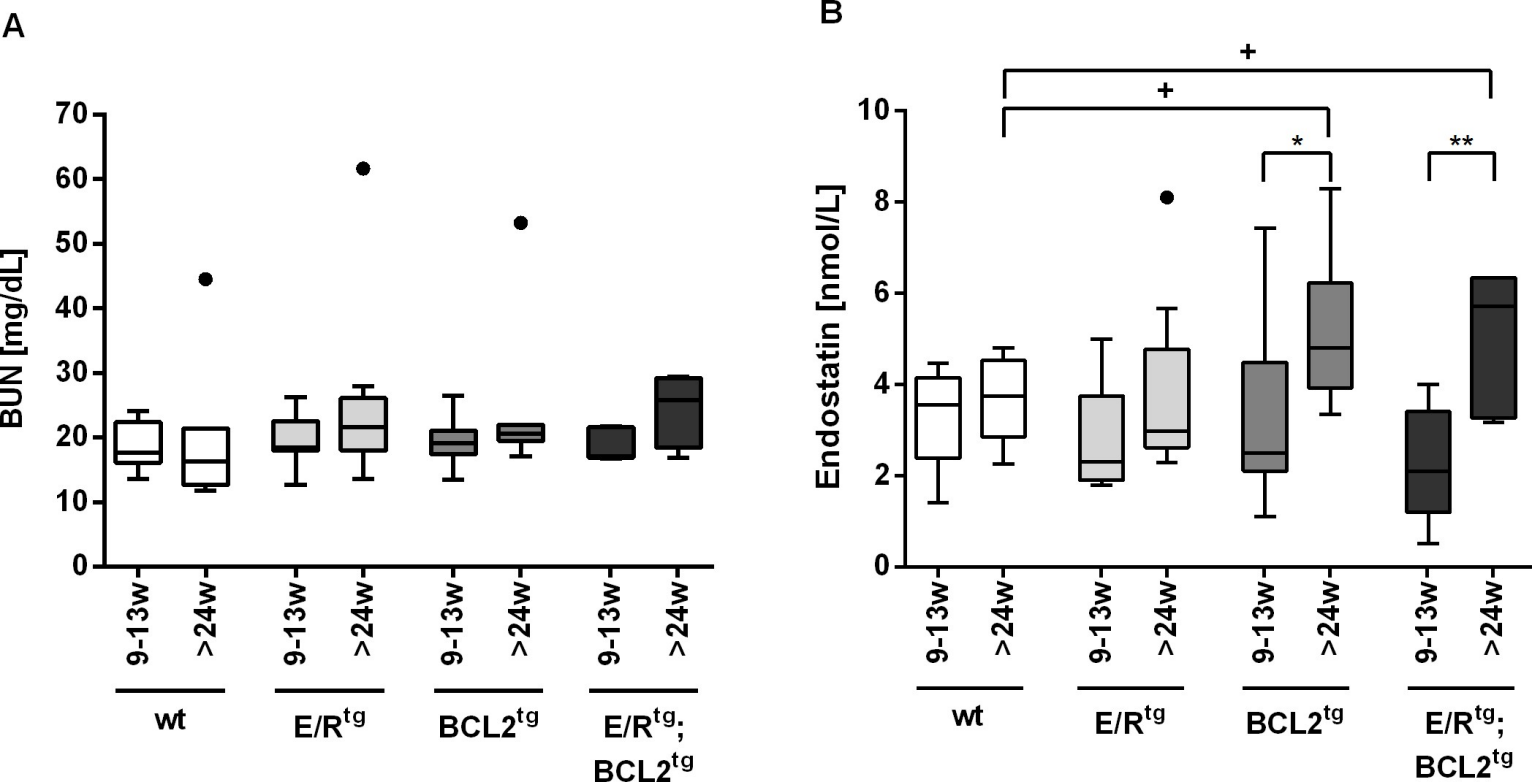
BUN and endostatin levels in young and older E/R^tg^, BCL2^tg^, double transgenic and control mice seem to display the kidney disease phenotype. Tukey’s boxplots indicate (A) blood urea nitrogen (BUN) levels (mg/dL) and (B) endostatin levels (nmol/L) in serum of 9–13 weeks and more than 24 weeks old wild type (wt), E/R^tg^, BCL2^tg^ and E/R^tg^;BCL2^tg^ mice. For BUN, n = 6, n = 8, n = 20, n = 5 for younger and n = 7, n = 11, n = 7 and n = 5 for older mice. For endostatin n = 8, n = 8, n = 19, n = 5 for younger and n = 10, n = 10, n = 8 and n = 6 for older mice. + indicates significance to wild type control (+ p<0.05) and * to young animals of same genotype with ** p<0.01 and * p<0.05.

Beside kidney pathophysiology BCL2^tg^ and double transgenic (E/R^tg^;BCL2^tg^) mice also displayed reduced renal function as addressed by significantly increased concentration of blood urea nitrogen (BUN) in terminally diseased mice [12]. Even before full-blown disease, the trend of elevated BUN levels in transgenic mice compared with wild type controls could be observed (Fig 5, S2 Table) [13].

Fig 5A shows mouse BUN concentration of all four genotypes at early (9–13 weeks) stage (mean wt 18.6, E/R^tg^ 19.4, BCL2^tg^ 19.2, E/R^tg^;BCL2^tg^ 18.8 mg/dL BUN). BUN levels in old (≥24 weeks) mice (mean wt 19.8, E/R^tg^ 24.7, BCL2^tg^ 24.7, E/R^tg^;BCL2^tg^ 24.2 mg/dL BUN) were clearly, but not significantly increased in all three transgenic mouse groups compared with younger animals of the same genotype and also compared with wild type controls. By contrast, mean endostatin concentration in mice with developed glomerulonephritis, i.e. old BCL2^tg^ and E/R^tg^;BCL2^tg^ animals (BCL2^tg^ 5.2, E/R^tg^;BCL2^tg^ 5.1 nmol/L endostatin) were significantly elevated compared with young mice (BCL2^tg^ 3.2, E/R^tg^;BCL2^tg^ 2.3 nmol/L endostatin). For wild type and E/R^tg^ animals without reduced kidney function, no difference could be observed (mean young wt 3.3, E/R^tg^ 2.8; old wt 3.7, E/R^tg^ 3.8 nmol/L endostatin).

**Fig 6:**
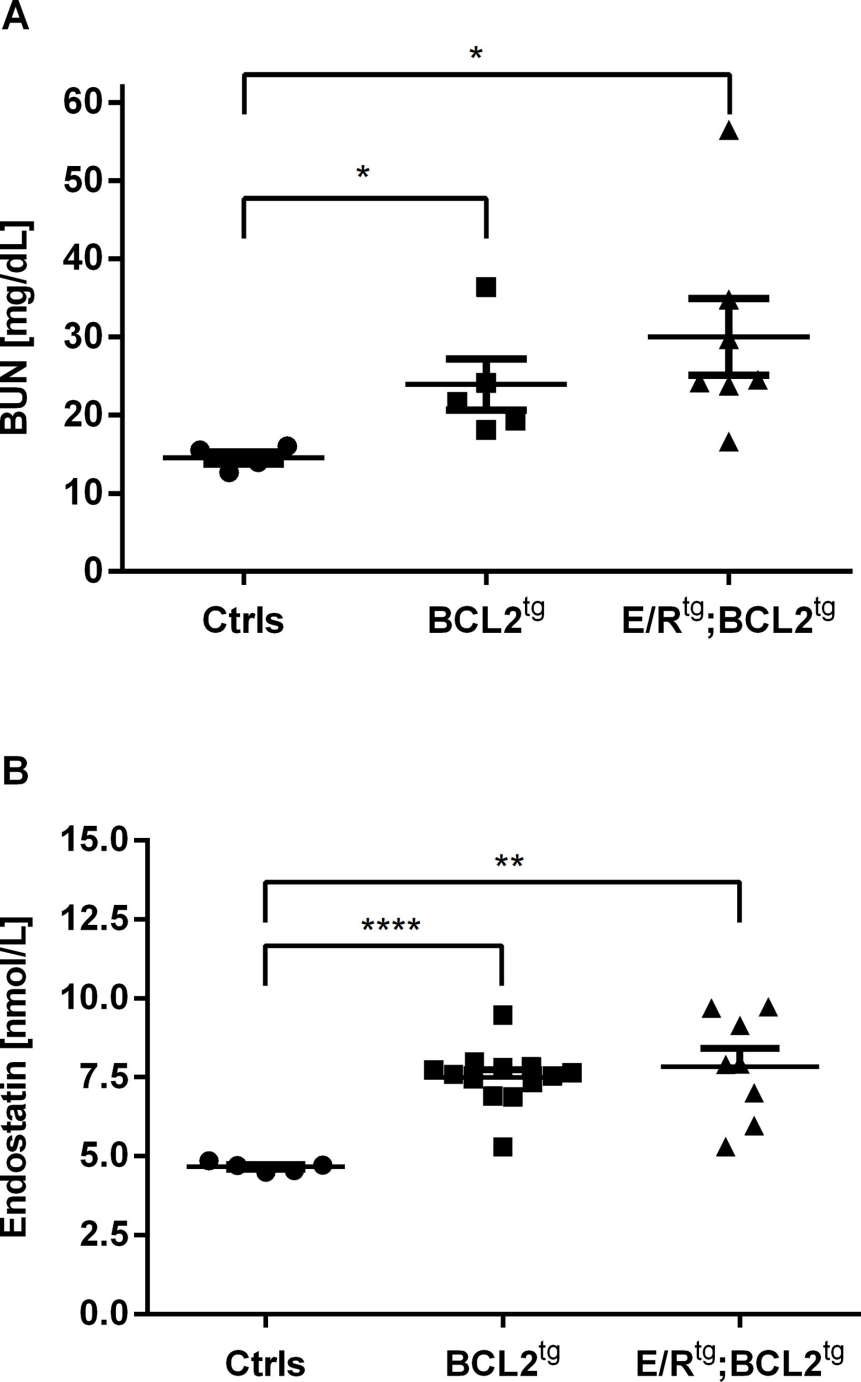
Increased BUN and endostatin levels of transplanted mice with impaired kidney function. (A) BUN (mg/dL) and (B) endostatin (nmol/L) in serum of immunocompromised NSG mice receiving cells from either control (ctrls) animals, or terminally diseased BCL2^tg^ or E/R^tg^;BCL2^tg^ mice, n = 4, n = 5 and n = 7 for BUN, n = 5, n = 13 and n = 8 for endostatin, respectively, measured 40 weeks after transplantation. Significance to controls is stated with **** p<0.0001, ** p<0.01 and * p<0.05.

Interestingly, when we transplanted bone marrow from terminally diseased mice (BCL2^tg^ and E/R^tg^;BCL2^tg^) and controls into immunocompromised NSG mice the recipient animals started to develop renal dysfunction as well. This phenotype is reflected by a significant increase in BUN levels (controls mean 14.6, BCL2^tg^ 23.9, E/R^tg^;BCL2^tg^ 30.0 mg/dL BUN) and endostatin (controls mean 4.7, BCL2^tg^ 7.5, E/R^tg^;BCL2^tg^ 7.8 nmol/L endostatin) concentration in the groups that received cells from BCL2^tg^ and double transgenic mice (Fig 6, S2 Table).

## Discussion

This study describes the validation and application of a specific, sample saving, reliable, rapid and robust sandwich ELISA for the detection of mouse and rat endostatin in complex serum and plasma samples. This assay was developed and validated to evaluate increased endostatin concentration as a potential new biomarker in mice with renal disease, and to compare endostatin with the established marker for nephropathy, blood urea nitrogen (BUN).

The assay has a standard range of 0-32 nmol/L with a lower limit of quantification of 0.5 nmol/L and a sensitivity of 0.24 nmol/L. These data are in line with the accessible data of the manufacturer. It could be shown here that the ELISA generates highly specific and therefore reliable signals for mouse and rat samples. Although it is a rapid assay (2.5 h) the outcome is not influenced by moderate changes of incubation temperature, reagent temperature or incubation time (drift over the plate). We also assessed dilution linearity and accuracy of the assay. Both parameters are comparable with the manufacturer (80–114% and 91–97%, respectively) and are within the range of acceptance, nevertheless there seem to be slight differences between the calibrator and endogenous endostatin. These marginal differences may occur because of the added C-terminal six His-tags to the recombinant calibrator protein. In this context, another modified recombinant human endostatin, known as Endostar, carries an N-terminal His-tag and was expressed in *E*. *coli*. China’s State Food and Drug Administration (SFDA) approved it for the treatment of non-small-cell lung carcinoma in 2005 [17]. Recombinant endostatin without His-tag was used in phase II clinical trials in the United States as well, but due to low potency, these trials failed. It is discussed, that the missing His-tag of the applied recombinant human endostatin led to impaired stability and therefore decreased potency [18]. Although the calibrator in the new mouse/rat endostatin assay has a C-terminal His-tag there could be an altered stability and slightly different behavior of the calibrator compared with endogenous endostatin, which might cause the variations in spike recovery and dilution linearity. The validation of this ELISA according to ICH and EMEA guidelines demonstrates that this assay can be used reliably and reproducibly for the detection of endostatin in mouse and rat samples and therefore may give new perspectives within the biomarker research.

There is no significant gender difference of endostatin concentration in serum of healthy humans, but a significant increase with age [19]. In line, our data obtained from mouse sera also only showed a slight trend of higher endostatin levels in male mice compared to female ones, but older mice displayed a trend of higher endostatin levels than younger ones.

Endostatin is one of the most potent anti-angiogenic factors. The anti-angiogenic activity of endostatin is not completely understood, but endostatin influences several molecular mechanisms like induction of endothelial cell apoptosis by reducing the expression of anti-apoptotic proteins like BCL-XL, BCL2 and BAD, inhibition of cell proliferation and cell migration and affecting the angiogenic signaling pathway [20].

Over the last two decades, many studies in rodents and humans revealed the association of elevated endostatin levels with several diseases, e.g. gastric cancer [21], diabetes mellitus [9], atherosclerosis [22], Alzheimer’s disease [23] and chronic kidney disease (CKD) [24]. Type XVIII collagen including its C-terminal fragment endostatin is one of the components in both glomerular and tubular basement membranes of the kidney. It could be shown recently that XVIII collagen deficient mice display loosening of the proximal tubular basement membrane, suggesting an essential role of XVIII collagen in the intact glomerular filtration barrier and tubular basement membrane structure [25]. Additionally, transgenic mice overexpressing endostatin revealed accelerated renal dysfunction and fibrosis in folic-acid induced nephropathy [10]. In accordance, we could show that the increase in serum endostatin concentration correlates with the severity of the glomerulonephritis phenotype of the BCL2^tg^and E/R^tg^;BCL2^tg^ double transgenic mice. This correlation with disease progression was even better than with the established kidney biomarker BUN. It is likely that the process by which endostatin is generated from type XVIII collagen in the kidney early on contributes to the pathogenesis of renal fibrosis and therefore elevated circulating endostatin levels might reflect the progression of renal disease earlier than BUN. Additionally, with impaired renal function urinary clearance of the 20 kDa endostatin may be further reduced, resulting in even higher endostatin serum concentration in these animals.

In humans Chen et al. have demonstrated that endostatin plasma levels in 201 CKD patients from the US were significantly higher compared to 201 controls without CKD and they found a correlation between the severity of disease and the concentration of endostatin [26]. Increasing endostatin might predict the progression of CKD and this was also shown in two independent longitudinal community-based cohorts of elderly subjects [5] and in patients with type 2 diabetes [9], where higher circulating endostatin concentrations were associated with increased risk of developing CKD. In all these studies focusing on endostatin levels and CKD, kidney disease was assessed by estimated GFR and/or albuminuria, but the correlation of endostatin levels with BUN concentration in humans is still missing. As we could show that the circulating level of endostatin in mice with a glomerulonephritis phenotype might be a better disease predictor than the established marker BUN, it would be of interest to further investigate endostatin as an important and early biomarker for reduced kidney function in a human cohort as well.

## Conclusion

The rigorous validation of this new commercially available mouse and rat endostatin ELISA shows that it meets all requirements for a robust, reliable and fast measurement of endostatin. This assay can be used in pre-clinical research for further investigations of the role of endostatin. We could show that increased endostatin concentration correlates with the glomerulonephritis phenotype in two mouse models and therefore we recommend endostatin as a useful and powerful biomarker for impaired kidney function in mice and rats. However, the role of endostatin in the development and progression of CKD remains to be determined and further studies in humans are needed.

## Supporting information

S1 ARRIVE The ARRIVE guidelines checklist for animal research/ in vivo experiments. (PDF)

S1 Table ELISA validation data used for Tables 1–6 and Figs 1–3. Endostatin concentration [nmol/L] and OD values of samples used for the measurement of specificity, cross-reactivity, intra- and inter-assay precision, limit of detection and lower limit of quantification, dilution linearity, accuracy, stability and robustness of the assay.
(XLSX)

S2 Table Data of endostatin and BUN measurement used for Figs 4–6. Concentration of endostatin [nmol/L] and BUN [mg/dL] in mice with different genotypes and age, as well as in mice, which underwent transplantation.
(XLSX)
